# Genome Sequence of the Thermotolerant Foodborne Pathogen *Salmonella enterica* Serovar Senftenberg ATCC 43845 and Phylogenetic Analysis of Loci Encoding Increased Protein Quality Control Mechanisms

**DOI:** 10.1128/mSystems.00190-16

**Published:** 2017-02-28

**Authors:** Scott V. Nguyen, Gregory P. Harhay, James L. Bono, Timothy P. L. Smith, Dayna M. Harhay

**Affiliations:** USDA ARS U.S. Meat Animal Research Center, Clay Center, Nebraska, USA; University of Delhi

**Keywords:** IncHI2, *Salmonella*, genome analysis, phylogenetic analysis, thermotolerance

## Abstract

*Salmonella enterica* subsp. *enterica* bacteria are important foodborne pathogens with major economic impact. Some isolates exhibit increased heat tolerance, a concern for food safety. Analysis of a finished-quality genome sequence of an isolate commonly used in heat resistance studies, *S. enterica* subsp. *enterica* serovar Senftenberg 775W (ATCC 43845), demonstrated an interesting observation that this strain contains not just one, but two horizontally acquired thermotolerance locus homologs. These two loci reside on a large 341.3-kbp plasmid that is similar to the well-studied IncHI2 R478 plasmid but lacks any antibiotic resistance genes found on R478 or other IncHI2 plasmids. As this historical *Salmonella* isolate has been in use since 1941, comparative analysis of the plasmid and of the thermotolerance loci contained on the plasmid will provide insight into the evolution of heat resistance loci as well as acquisition of resistance determinants in IncHI2 plasmids.

**IMPORTANCE** Thermal interventions are commonly used in the food industry as a means of mitigating pathogen contamination in food products. Concern over heat-resistant food contaminants has recently increased, with the identification of a conserved locus shown to confer heat resistance in disparate lineages of Gram-negative bacteria. Complete sequence analysis of a historical isolate of *Salmonella enterica* serovar Senftenberg, used in numerous studies because of its novel heat resistance, revealed that this important strain possesses two distinct copies of this conserved thermotolerance locus, residing on a multireplicon IncHI2/IncHI2A plasmid. Phylogenetic analysis of these loci in comparison with homologs identified in various bacterial genera provides an opportunity to examine the evolution and distribution of loci conferring resistance to environmental stressors, such as heat and desiccation.

## Abstract (precis)

Thermal interventions are commonly used in the food industry as a means of mitigating pathogen contamination in food products. Concern over heat-resistant food contaminants has recently increased, with the identification of a conserved locus shown to confer heat resistance in disparate lineages of Gram-negative bacteria. Complete sequence analysis of a historical isolate of *Salmonella enterica* serovar Senftenberg, used in numerous studies because of its novel heat resistance, revealed that this important strain possesses two distinct copies of this conserved thermotolerance locus, residing on a multireplicon IncHI2/IncHI2A plasmid. Phylogenetic analysis of these loci in comparison with homologs identified in various bacterial genera provides an opportunity to examine the evolution and distribution of loci conferring resistance to environmental stressors, such as heat and desiccation.

## INTRODUCTION

Heat-resistant bacterial contaminants have increasingly become a source of concern in the last several years especially with regard to food safety and human health. Although many members of the family *Enterobacteriaceae* exhibit variable heat resistance after heat shock, relatively few strains have been found to demonstrate an innate thermotolerant phenotype. Recently, however, a genetic locus found to confer heat resistance has been identified in certain strains of *Cronobacter sakazakii*, *Escherichia coli*, and *Klebsiella* species (1–3). Using a top-down proteomic approach, Williams et al. identified a protein biomarker sequence in *Cronobacter* correlating with thermotolerance to reverse engineer the DNA sequence of the protein that was homologous to a protein found in thermotolerant *Methylobacillus flagellatus* (4). Gajdosova and coworkers expanded upon this work and sequenced an 18-kbp region containing this open reading frame (ORF) and determined that the full locus is required for highest thermotolerance (1). Independently, Bojer et al. (3) isolated several *Klebsiella pneumoniae* strains over the course of a 2-year surveillance study of patients in a Danish hospital; this study demonstrated multiple antibiotic resistance phenotypes (3). These nosocomial isolates were associated with persistence in contaminating reusable endoscopes, and Bojer et al. (3) hypothesized that the adaptability of the isolates was due to their resiliency to harsh environmental stressors such as heat. Bojer et al. then identified a locus encoding small heat shock proteins and a novel Clp ATPase termed ClpK (3). This locus showed high similarity to the same chromosomal region from *Methylobacillus* that Williams et al. (4) identified and Gajdosova later sequenced (1). Subsequently, Bojer and coworkers reported that the *clpK* gene was cotransferred with plasmids encoding multiple drug resistance hosted by emerging *K. pneumoniae* clones associated with nosocomial outbreaks (5). More recently, Mercer et al. (2) and Dlusskaya et al. (6) sequenced an *E. coli* beef isolate that was able to survive in beef patties grilled to 71°C. Genomic comparisons of highly heat-resistant *E. coli* isolates resulted in the identification of a 14-kbp locus of heat resistance with greater than 99% identity to the aforementioned loci in *Cronobacter* and *Klebsiella* (2). Lee et al. expanded on the thermotolerance genetic module, noting that other ORFs on the loci are homologous to proteins involved in protein homeostasis and proposed that the loci be called transmissible loci for protein quality control (TLPQC) (7, 8). This locus appears to have been horizontally acquired in some bacterial lineages, as it is found not only in a number of clinically relevant members of the family *Enterobacteriaceae* but also more broadly in other *Proteobacteria* species (7).

A striking example of innate thermotolerance in the foodborne pathogen *Salmonella enterica* subsp. *enterica* has been reported for a particular strain of *S*. *enterica* serovar Senftenberg (9). This isolate, *S*. Senftenberg ATCC 43845 (originally referred to as strain 775W), has been used extensively in thermal resistance studies over the last several decades (9–12); it was first reported to be extremely thermotolerant in 1946 by Winter et al. (13), having been isolated previously by E. Beckler in 1941 from Chinese egg powder at the Massachusetts Department of Public Health (14). While dried egg products are not commonly sold in modern times, dried egg powder was used in rations in many Western countries, especially England, during the onset of World War II. Indeed, multiple reports of salmonellosis in the 1940s were associated with consumption of powdered eggs (15, 16).

**FIG 1:**
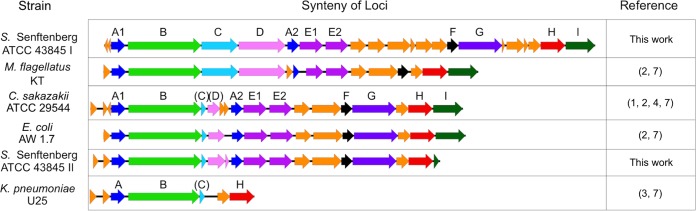
Genetic synteny is highly conserved between TLPQC (transmissible loci for protein quality control). The synteny of the TLPQC is highly conserved, suggesting an origin of horizontal gene transfer between several genera (*Salmonella*, *Methylobacillus*, *Cronobacter*, *Escherichia*, and *Klebsiella*). Despite reductions in the loci, the synteny of the loci is conserved in the various clades. Conserved ORFs with predicted functions are colored in the figure. The functions are indicated by the following letters (and colors): A, small heat shock proteins (dark blue); B, Clp protease (green); C, cardiolipin synthase (teal); D, FtsH protease (pink); E, YfdX family protein (magenta); F, thioredoxin (black); G, KefB glutathione-regulated potassium efflux pump (purple); H, Zn-dependent protease (red); and I, periplasmic serine protease (dark green). Letters within parentheses indicate that the gene is truncated. The *E. coli* AW 1.7 nucleotide accession number is NZ_LDYJ01000141.

Despite being used in numerous studies examining thermotolerance, the genetic determinants conferring heat resistance in *S*. Senftenberg ATCC 43845 have not been identified previously. Analysis of the complete genome of *S*. Senftenberg ATCC 43845 revealed the presence of two closely related loci with more than 98% identity to the thermotolerance loci previously studied in *Cronobacter*, *Escherichia*, *Klebsiella*, and* Pseudomonas* (1–3, 8) on a potentially conjugable IncHI2 plasmid. As the synteny of the genetic loci is highly conserved broadly across diverse members of the phylum *Proteobacteria*, here we designate the putative thermotolerance loci in *S*. Senftenberg TLPQC-1 and TLPQC-2 (Fig. 1) in keeping with the nomenclature proposed by Lee et al. (7). Comparative analysis of the *S*. Senftenberg TLPQCs, as well as other known homologs, provides a unifying framework for understanding the origin and evolution of loci conferring mechanisms of increased protein maintenance and reveals the prevalence and conservation of these loci within the *Proteobacteria*.

## RESULTS AND DISCUSSION

### Genomic features of *S*. Senftenberg ATCC 43845.

**FIG 2:**
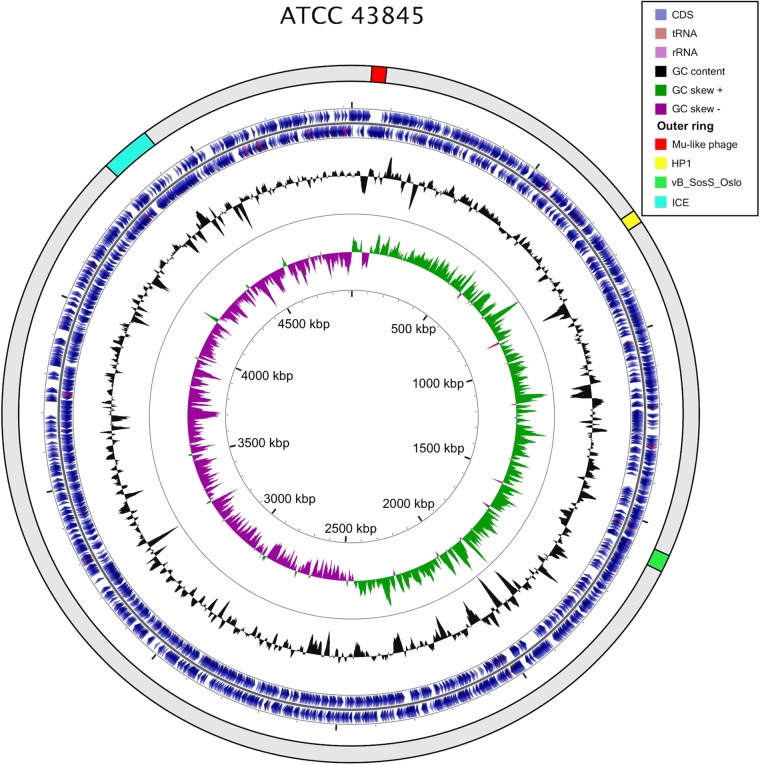
Genome map of *S*. Senftenberg ATCC 43845. The outer ring displays the mobile genetic elements found in *S*. Senftenberg ATCC 43845. The second and third rings depict predicted CDS on the forward and reverse strands, respectively. The innermost rings depict GC content and skew.

S. Senftenberg ATCC 43845 has been used extensively in thermal resistance studies over the last several decades, having been identified as an extremely thermotolerant strain of *Salmonella* in 1946 by Winter et al. (13). A draft genome assembly consisting of 114 contigs (139-kb contig N50; accession number NZ_AOXX00000000) and a total length of 5,184,857 bases have previously been reported for this strain (17). Here we present the complete and closed, finished-quality genome sequence of *S*. Senftenberg ATCC 43845 based on long-read sequencing. The circularized chromosome consists of 4,920,660 bp with a chromosomal GC content of 52.2%, comparable to other finished *Salmonella* (Fig. 2). Annotation by the NCBI Prokaryotic Genome Annotation Pipeline (version 3.3) indicated the presence of 5,105 total genes, with 4,980 coding sequences (CDS) and 125 genes encoding RNAs (GenBank accession number CP016837). This includes 3,930 predicted proteins with homology to known proteins, in addition to 979 hypothetical or “miscellaneous” predicted proteins. In addition, the chromosome contains potential restriction-modification (RM) systems, including a DNA (cytosine-5)-methyltransferase motif, as well as phage integration-related N^6^-adenine methylation systems. Genome analysis using PHASTER (18) revealed the presence of two intact prophages and one questionable prophage (Fig. 2). Manual inspection revealed a Mu-like prophage, with up to 77% nucleotide identity but only 11% coverage of phage D108 by PHASTER at nucleotide positions 30,172 to 82,470. The Mu-like prophage is integrated downstream of *mnmE* (also known as *thdF* or *trmE*) which is a hot spot integration site for other mobile genetic elements in *Salmonella* (19). The other intact phage identified by PHASTER is a prophage related to the *Haemophilus influenzae* HP1 phage at positions 738,135 to 768,638. Manual inspection of the phage region identified as questionable by PHASTER analysis showed what appears instead to be an intact prophage related to the *Salmonella* phage vB_SosS_Oslo at nucleotides 1,549,983 to 1,599,675. No known virulence factors were associated with any of the prophages. A putative integrative conjugative element (ICE) identified by BLASTn is integrated into tRNA^Phe^ at nucleotide positions 4,324,610 to 4,433,343. BLAST analysis of the ICE showed no virulence factors; however, the element appears to encode an arsenic resistance locus and a copper homeostasis and silver resistance island (CHASRI) (20). Significantly, there were no predicted thermotolerance loci on the chromosome of the isolate revealed by BLAST analysis.

### Features of the pSSE ATCC-43845 plasmid.

**FIG 3:**
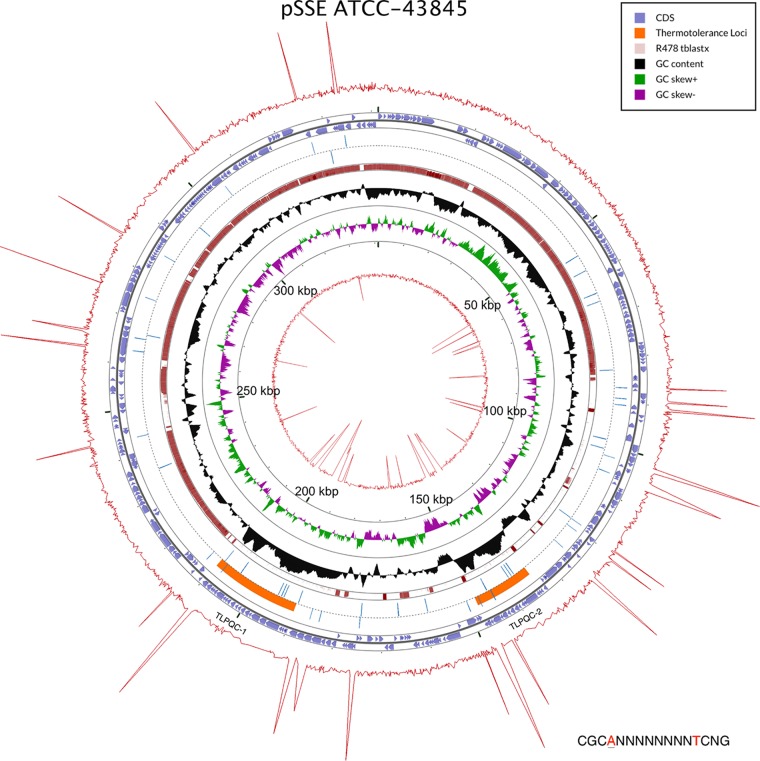
Map of the pSSE ATCC-43845 plasmid. The thermotolerance islands are indicated as orange regions on the map and demonstrate increased high GC content (black track). pSSE ATCC-43845 shares high homology with the backbone of the R478 plasmid (red tBLASTx). Base modification signals generated by BaseModFunctions v2.1.R and Circos are overlaid on the plasmid map. Qmod values are height proportional to the interpulse distance ratio for each of the modified bases on the positive strand (red outermost track) and negative strand (red innermost track). Blue hash marks correspond to the CGCAN_8_TCNG motifs present on the plasmid.

The finished assembly of *S*. Senftenberg ATCC 43845 revealed the presence of a large 341,373-bp circular plasmid, not previously described for this strain, and is here designated pSSE ATCC-43845. Combining the plasmid length with the chromosome takes the total genome complement of the strain to 5,262,033 bases, slightly exceeding that predicted by the short-read draft assembly (17). Annotation of this plasmid sequence via the same method as the chromosome predicts 336 genes on the plasmid, including two complete thermotolerance loci with distinct sequences between nucleotides 189,272 to 211,036 and 134,033 to 148,118 of GenBank accession number CP016838 (Fig. 3). These two loci are designated TLPQC-1 and TLPQC-2. Both thermotolerance islands have characteristic high average GC content (61%), contain the canonical *clp* protease gene, and are flanked by small heat shock proteins and predicted DNA-binding genes, in keeping with previously reported heat tolerance islands (Fig. 1) (1–3, 7). The GC content of the islands suggests that the origins of TLPQC-mediated heat resistance lie in *Proteobacteria* with higher average GC content than *Salmonella* species.

**FIG 4:**
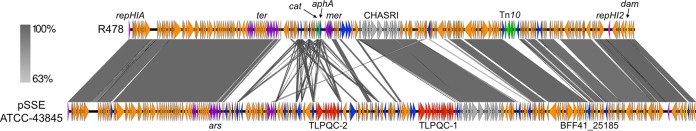
BLASTN comparison of the R478 and pSSE ATCC-43845 plasmids. The backbone for R478 is highly conserved in pSSE ATCC-43845. The majority of differences between the two plasmids reside between the tellurite resistance island and CHASRI (copper homeostasis and silver resistance island). The lack of any known antibiotic resistance genes, such as *aphA* (kanamycin) or *cat* (chloramphenicol), on pSSE ATCC-43845 is also noted. The integration site for Tn*10* with tetracycline resistance is in BFF41_25185 in pSSE ATCC-4384. Heavy metal resistance genes (purple), antibiotic resistance genes (teal), TLPQC genes (red), transposase genes (blue), CHASRI genes (silver), and *rep* (magenta) and the mercury resistance (*mer*), methylase (*dam*), tellurite resistance (*ter*), arsenic resistance (*ars*), and replicase (*rep*) genes are indicated.

BLASTn comparison of the entire plasmid sequence to microbial sequences in GenBank indicates that it is closely related to the lncHI2 R478 plasmid, sharing up to 98% identity with more than 61% coverage. *In silico* analysis of the plasmid sequence with PlasmidFinder (21) identified two replicon regions, *IncHI2* and *IncHI2A*. Comparative analyses further revealed that the pSSE ATCC-43845 plasmid has a comparable GC content value of 48.0% to the GC content of R478 at 45.5% (Fig. 3) (22, 23). Further evidence that pSSE ATCC-43845 is a member of the IncHI2 plasmid family is found by comparing transposon insertion sites which have been used as markers to track closely related plasmids of the same family. In R478 and closely related IncHI2 plasmids with antibiotic resistance, Tn*10* and the linked tetracycline resistance genes have been used for this purpose (24), and in pSSE ATCC-43845, the Tn*10* transposition site (BFF41_25185 [Fig. 4]) was found to be intact. As with R478, several heavy metal resistance determinants are present on pSSE ATCC-43845, including a tellurite resistance island and another copy of CHASRI (23, 25). As illustrated in Fig. 4, the variable regions of R478 and pSSE ATCC-43845 are bracketed by the tellurite resistance operon and CHASRI. This variable region in R478 encodes mercury resistance and chloramphenicol (*cat*) and kanamycin (*aphA*) resistance, while in pSSE ATCC-43845, this region contains the two TLPQC loci. DNA sequence analyses showed that pSSE ATCC-43845 does not contain genes encoding drug resistance determinants. In keeping with this finding, antimicrobial sensitivity phenotyping (as evaluated by broth microdilution testing methods; TREK, Sensititre CMV3AGNF plates) showed *S*. Senftenberg ATCC-43845 to be pansusceptible to 15 antimicrobial agents tested on the panel. This finding is novel and suggests that pSSE ATCC-43845 is a relic of IncHI2 plasmids before the era of therapeutic antibiotic use, as present-day examples of IncHI2 plasmids have frequently been identified as carriers of antibiotic resistance genes (26–28).

### Methylation analysis.

Sequence analysis using the Pacific Biosciences platform produces data that can detect base modification in the source DNA (29). Detecting these modifications is dependent on both the depth of sequence coverage and the magnitude of the effect of the modification on the kinetics of nucleotide incorporation. Nucleotide modification is predicted from the interpulse distance data (see Materials and Methods) with N^6^-adenine methylation producing a strong signal that is easily detected at low coverage, while N^5^-cytosine methylation demonstrates a weaker signal that requires deeper coverage. Methylation motif analysis of the *S*. Senftenberg sequence data and comparisons to established motifs in REBASE (30) showed adenine N^6^-methylation of the sites GATC, CAGAG, and ATGCAT (the adenine methylated is shown underlined), as observed previously with other *Salmonella* serovars (31). Additional motifs were identified in *S*. Senftenberg and are summarized in Table 1. Of particular interest was methylation of the CGCAN_8_TCNG motif, as the presence of a cluster of three of these motifs was observed in each of the novel *clp* protease genes found in TLPQC-1 and TLPQC-2. This is illustrated in Fig. 3, where modification values (Qmod) indicating methylation of this site on the positive and negative strands are visualized as the outermost and innermost tracks, respectively, on pSSE ATCC-43845. Depiction of the methylation state of this site on the plasmid also reveals a skew of the presence of these motifs, which is likely due to the chimerism of the R478 backbone. The presence of these motifs in TLPQC-1 and -2 is noteworthy and hints at the possibility that this motif may play some regulatory role in the expression of genes contained in the TLPQC loci. Additional base modification motif maps of the plasmid are visualized in the supplemental figures (see Fig. S1 and S2 in the supplemental material).

10.1128/mSystems.00190-16.1 FIG S1  GATC methylation for pSSE ATCC-43845. The map of plasmid pSSE ATCC-43845 is shown, with the thermotolerance locus islands indicated as orange regions. Base modification signals generated by BaseModFunctions v2.1.R and Circos are overlaid on the plasmid map. Qmod values are height proportional to the interpulse distance ratio for each of the modified bases on the positive strand (red outmost track) and negative strand (red innermost track). Blue hash marks correspond to the GATC motifs present on the plasmid. Download FIG S1, PDF file, 2.2 MB. Copyright © 2017 Nguyen et al. 2017 Nguyen et al. This content is distributed under the terms of the Creative Commons Attribution 4.0 International license.

10.1128/mSystems.00190-16.2 FIG S2  CAGAG methylation for pSSE ATCC-43845. The map of plasmid pSSE ATCC-43845 is shown, with the thermotolerance locus islands indicated as orange regions. Base modification signals generated by BaseModFunctions.v.2.1.R and Circos are overlaid on the plasmid map. Qmod values are height proportional to the interpulse distance ratio for each of the modified bases on the positive strand (red outmost track) and negative strand (red innermost track). Green hash marks correspond to the CAGAG motifs present on the plasmid. Download FIG S2, PDF file, 1.6 MB. Copyright © 2017 Nguyen et al. 2017 Nguyen et al. This content is distributed under the terms of the Creative Commons Attribution 4.0 International license.

**TABLE 1:** Base modification summary table

Motif^a^	MTase ORF^b^	No. of motifs in genome^c^	No. of motifs modified^c^	% modified^c^	Type/subtype^d^	Coverage^e^
CAG**A**G	BFF41_17760	6,607	6,576	99.5	III beta	82.9
A**T**GC**A**T	BFF41_02695	776	248	37.7//37.7	II beta	88.8
**A**CYN_6_G**T**TC	BFF41_00175	797//797	793//796	99.5//99.9	I gamma	80.6
CGC**A**N_8_**T**CNG^f^	BFF41_20045	849//849	847//838	99.8//98.7	I gamma	82.3
GA**A**N_7_R**T**AC^f^	BFF41_21025	1,036//1,036	1,019//1,030	98.4//99.4	I gamma	82.0
G**AT**C^g^	BFF41_02205	41,312	41,202	99.7//99.7	II	82.6

a Modified adenine bases are shown in bold type; cognate bases are shown in bold font.

b Motifs are assigned to their respective putative methylases (methylase [MTase] ORFs).

c Numbers separated by double slashes indicate the values for motifs detected on the positive and negative strand shown before and after the double slashes, respectively, if known.

d The type and subtype refer to the class of methylase.

e Coverage indicates the depth of sequence at the minimum quality threshold set in the protocol.

f Motifs determined to be unique to *S*. Senftenberg ATCC 43845, as determined by REBASE.

g The G**AT**C motif cannot be assigned unambiguously, but BFF41_02205 was assigned as the most likely candidate.

### Comparison of TLPQC loci.

**FIG 5:**
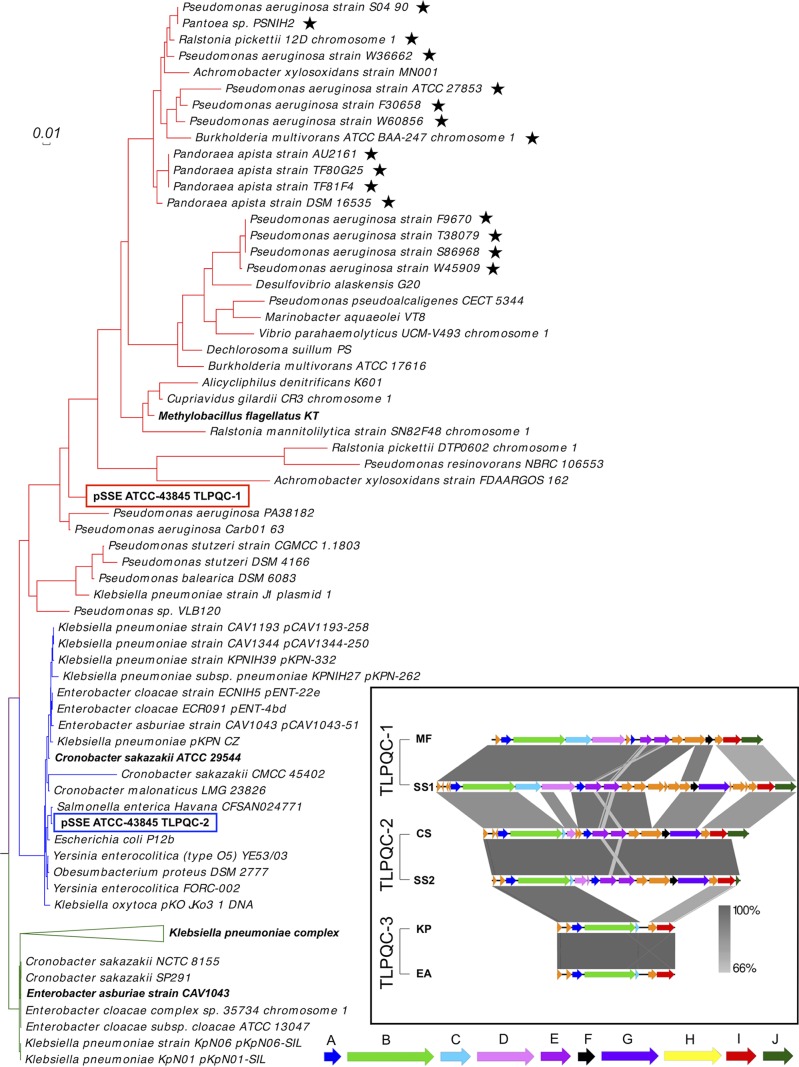
Genomic comparison of thermotolerance islands. MrBayes phylogeny tree reveals three different clades for TLPQC-1 (red branches), TLPQC-2 (blue branches), and TLPQC-3 (green branches). Graphic representations of representatives indicated in bold along the tree are shown to the right in the inset to illustrate conservation, with gray connections between the loci indicating sequence homology. The thermotolerance islands of *Proteobacteria* show high degrees of conservation, indicating an origin of horizontal gene transfer. Very high genetic conservation is observed within the clade, with less conservation between clades. The truncated cardiolipin synthase and FtsH CDS is conserved across all members of TLPQC-2. Strains in the inset are as follows: *Methylobacillus flagellatus* (MF), *S*. Senftenberg TLPQC-1 (SS1), *Cronobacter sakazakii* (CS), *S*. Senftenberg TLPQC-2 (SS2), *Klebsiella pneumoniae* (KP), and *Enterobacter asburiae* (EA). The functions of the genes are indicated by the following letters (and colors): A, small heat shock proteins (dark blue); B, Clp protease (green); C, cardiolipin synthase (teal); D, FtsH protease (pink); E, YfdX family protein (magenta); F, thioredoxin (black); G, KefB glutathione-regulated potassium efflux pump (purple); H, conserved protein with GGDEF domain (yellow); I, Zn-dependent protease (red); J, periplasmic serine protease (dark green). CDS in orange indicate hypothetical proteins. Black stars indicate TLPQCs that are found within bioinformatically identified integrative conjugative elements.

A phylogenetic analysis (see Materials and Methods) was performed to determine the relationship of the *Salmonella* heat tolerance islands to those in *Klebsiella* plasmids, *Pseudomonas* chromosomes, and other bacteria. Heat tolerance islands were identified by searching the literature (previous reports) or by searching for similarity of nucleotide sequence records in GenBank to representative TLPQC sequences by BLASTn (see Materials and Methods). A total of 91 heat tolerance islands, including 48 reported to be located on chromosomes, 42 reported to reside on plasmids, and one presumably on a plasmid (Table 2), as well as the two TLPQC islands from plasmid pSSE ATCC-43845, were included in the analysis. For each species, the thermotolerance island was manually curated from the reported sequence (Table 2). Previously, two clades (A and B) of heat tolerance islands, which were termed “locus of heat resistance” (LHR), were identified (2). We chose to use instead the nomenclature of TLPQC proposed and adopted by Lee et al. (7), since a number of ORFs within the LHR islands have been shown or predicted to encode protein quality control functions involved in surviving a range of stressors beyond heat, including antibiotic exposure, desiccation, and oxidative stress. However, we propose to amend the TLPQC classification by Lee et al. (7) and incorporate phylogenetic analysis from our work and Mercer et al. (2) for multiple clades (Fig. 5). Thus, the broader phylogeny incorporating the pSSE ATCC-43845 islands (Fig. 5) indicates three clades, TLPQC-1, -2, and -3, with the third clade comprised of a reduced TLPQC island primarily observed in plasmids of *Klebsiella*. There is high genetic conservation within clades and less genetic conservation between clades, and genetic synteny is maintained across multiple genera (Fig. 1 and 5). The two pSSE ATCC-43845 islands fall within clades 1 and 2, consistent with the names assigned here, and are no more closely related to other plasmid-borne islands than to chromosome-borne islands.

**TABLE 2:** Strain information for the sequence data used in the phylogenetic analysis of TLPQC loci in this study

Species	Strain or plasmid	Accession no.	TLPQC^a^	Location
*Achromobacter xylosoxidans*	FDAARGOS_162	CP014065	1	Chromosome
	MN001	CP012046	1	Chromosome
*Alicycliphilus denitrificans*	K601	CP002657	1	Chromosome
*Burkholderia multivorans*	ATCC 17616	CP000868	1	Chromosome
*Burkholderia multivorans* chromosome 1	ATCC BAA-247	CP009832	1	Chromosome
*Cupriavidus gilardii* chromosome 1	CR3	CP010516	1	Chromosome
*Dechlorosoma suillum*	PS	CP003153	1	Chromosome
*Desulfovibrio alaskensis*	G20	CP000112	1	Chromosome
*Klebsiella pneumoniae*	J1 plasmid 1	CP013712	1	Plasmid
*Marinobacter aquaeolei*	VT8	CP000514	1	Chromosome
*Methylobacillus flagellatus*	KT	CP000284	1	Chromosome

*Pandoraea apista*	AU2161	CP011501	1	Chromosome
	DSM 16535	CP013481	1	Chromosome
	TF80G25	CP011279	1	Chromosome
	TF81F4	CP010518	1	Chromosome
*Pantoea* sp.	PSNIH2	CP009866	1	Chromosome

*Pseudomonas aeruginosa*	ATCC 27853	CP015117	1	Chromosome
	F30658	CP008857	1	Chromosome
	F9670	CP008873	1	Chromosome
	PA38182	HG530068	1	Chromosome
	S04 90	CP011369	1	Chromosome
	S86968	CP008865	1	Chromosome
	T38079	CP008866	1	Chromosome
	W36662	CP008870	1	Chromosome
	W45909	CP008871	1	Chromosome
	W60856	CP008864	1	Chromosome
	Carb01 63	CP011317	1	Chromosome

*Pseudomonas balearica*	DSM 6083	CP007511	1	Chromosome
*Pseudomonas pseudoalcaligenes*	CECT 5344	HG916826	1	Chromosome
*Pseudomonas resinovorans*	NBRC 106553	AP013068	1	Chromosome
*Pseudomonas* sp.	VLB120	CP003961	1	Chromosome
*Pseudomonas stutzeri*	CGMCC 1.1803	CP002881	1	Chromosome
	DSM 4166	CP002622	1	Chromosome
*Ralstonia mannitolilytica* chromosome 1	SN82F48	CP010799	1	Chromosome
*Ralstonia pickettii* chromosome 1	12D	CP001644	1	Chromosome
	DTP0602	CP006667	1	Chromosome
*Salmonella enterica* Senftenberg	pSSE ATCC-43845	CP016838	1	Plasmid
*Vibrio parahaemolyticus* chromosome 1	UCM-V493	CP007004	1	Chromosome
*Cronobacter malonaticus*	LMG 23826	CP013940	2	Chromosome
	CMCC 45402	CP006731	2	Chromosome
*Cronobacter sakazakii*	ATCC 29544	CP011047	2	Chromosome
*Enterobacter asburiae*	CAV1043 pCAV1043-51	CP011587	2	Plasmid
*Enterobacter cloacae*	ECNIH5 pENT-22e	CP009855	2	Plasmid
	ECR091 pENT-4bd	CP008907	2	Plasmid
*Escherichia coli*	P12b	CP002291	2	Chromosome
*Klebsiella oxytoca*	pKO_JKo3_1 DNA	AP014952	2	Plasmid
*Klebsiella pneumoniae*	CAV1193 pCAV1193-258	CP013323	2	Plasmid
	CAV1344 pCAV1344-250	CP011623	2	Plasmid
	KPNIH39 pKPN-332	CP014763	2	Plasmid
	pKPN_CZ	JX424424	2	Plasmid

*Klebsiella pneumoniae* subsp. *pneumoniae*	KPNIH27 pKPN-262	CP007734	2	Plasmid
*Obesumbacterium proteus*	DSM 2777	CP014608	2	Chromosome
*Salmonella enterica* Havana	CFSAN024771	JWQI01000019.1	2	Unknown
*Salmonella enterica* Senftenberg	pSSE ATCC-43845	CP016838	2	Plasmid
*Yersinia enterocolitica*	(Type O:5) YE53/03	HF571988	2	Chromosome
	FORC-002	CP009456	2	Chromosome
*Cronobacter sakazakii*	SP291	CP004091	3	Chromosome
	NCTC 8155	CP012253	3	Chromosome
*Enterobacter asburiae*	CAV1043	CP011591	3	Chromosome
*Enterobacter cloacae* complex chromosome 1	35734	CP012162	3	Chromosome
*Enterobacter cloacae* subsp. *cloacae*	ATCC 13047	CP001918	3	Chromosome

*Klebsiella pneumoniae*	207M1D0 KPN207_p3	LT216439	3	Plasmid
	30660/NJST258_1 pNJST258N1	CP006927	3	Plasmid
	32192p	CP010574	3	Plasmid
	34618 p34618	CP010393	3	Plasmid
	500_1420 p500_1420	CP011981	3	Plasmid
	BK32179 pBK32179	JX430448	3	Plasmid
	CAV1392 pCAV1392-131	CP011577	3	Plasmid
	DMC1097 pDMC1097	CP011977	3	Plasmid
	JM45 p1	CP006657	3	Plasmid
	KP-1 pKP1-19	CP012884	3	Plasmid
	KpN01 pKpN01-SIL	CP012989	3	Plasmid
	KpN06 pKpN06-SIL	CP012994	3	Plasmid
	Kpn555 pKPN-d90	CP015132	3	Plasmid
	KPNIH36 pKPN-fff	CP014649	3	Plasmid
	O6CO7 pIT-6C07	LT009688	3	Plasmid
	pKN-LS6	JX442974	3	Plasmid
	pKp848CTX	LM994717	3	Plasmid
	Plasmid 1	CP015823	3	Plasmid
	PMK1 PMK1-A	CP008930	3	Plasmid
	pUUH239.2	CP002474	3	Plasmid
	ST15 pKP02022	KF719972	3	Plasmid
	ST23 pKP007	KF719971	3	Plasmid
	ST258 pKPN-IT	JN233704	3	Plasmid
	ST48 pKP09085	KF719970	3	Plasmid
	U25 PU25001	KT203286	3	Plasmid
	UHKPC07 pUHKPC07	CP011987	3	Plasmid
	UHKPC33 pUHKPC33	CP011990	3	Plasmid

*Klebsiella pneumoniae* subsp. *pneumoniae*	KPNIH10 pKPN-498	CP007729	3	Plasmid
	KPNIH24 pKPN-e44	CP008800	3	Plasmid
	KPNIH32 pKPN-a68	CP009777	3	Plasmid
	KPX pKPX-1	AP012055	3	Plasmid
	MGH 78578 pKPN3	CP000648	3	Plasmid

a The TLPQC-1, TLPQC-2, and TLPQC-3 loci are indicated by 1, 2, and 3, respectively, in the table.

Members of the TLPQC-1 clade are broadly distributed within the *Proteobacteria* (Fig. 5) with examples found in environmental isolates such as *Methylobacillus flagellatus* and *Dechlorosoma suillum* (32, 33), as well as within clinically relevant bacteria such as *Pseudomonas*, *Pandorea*, and *Burkholderia* (8). The majority of TLPQC-1 were found to be chromosomally located, with the exception of *S*. Senftenberg TLPQC-1 and another on plasmid 1 of *Klebsiella pneumoniae* J1 (GenBank accession number CP013712). Comparative analyses of these chromosomally bound TLPQCs suggest that they are inserted into larger mobile genetic elements (data not shown). TLPQC-1 genes in strains marked with black stars (Fig. 5) indicate that the loci are found within an integrative conjugative element (ICE) that is integrated into tRNA^Gly^. Attachment repeats flanking the putative ICE were identified with the core *att* nucleotide sequence 5′ TGGAGCGGGCGATGGGAA. Members of the TLPQC-1 lineage contain the longest islands of the three lineages (~15-kbp average), with core genes predicted to encode a phage-like transcriptional regulator, two small heat shock protein variants, a novel Clp protease, cardiolipin synthase, the membrane-associated ATP-dependent protease FtsH, two variants of YfdX protein family with potential chaperone activity, thioredoxin, a glutathione regulated K^+^ efflux pump related to KefB, a phosphate starvation-inducible PsiE family protein, a Zn-dependent protease, and a periplasmic serine protease (7) (Fig. 5).

Although most of the ORFs in TLPQC-1 have not been experimentally verified, one of the small heat shock proteins, sHsp20c, was recently characterized and found to aggregate in a 24-mer that plays a role in ameliorating stress resistance as a protein chaperone in *Pseudomonas* (8). Additionally, the novel Clp protease, ClpG_G1_, has been shown to confer thermal resistance in *Cronobacter*, *Escherichia*, *Pseudomonas*, and other *Enterobacteriaceae* (3, 8). The rest of the ORFs in TLPQC-1 have not been studied in depth; however, it has been shown that the full TLPQC is needed for the greatest thermal protection (1). The conservation of TLPQC and protein domains within the uncharacterized ORFs to known proteases, chaperones, and other quality control proteins suggests that these additional ORFs may contribute to survival in other adverse conditions such as osmotic shock or desiccation resistance.

The second lineage observed, TLPQC-2, is the best-studied TLPQC thus far, having first been identified in *Cronobacter sakazakii* contaminants of powdered infant formula (1). Islands in this lineage are shorter than TLPQC-1 (~14 kb) primarily because of the truncation of genes predicted to encode cardiolipin synthase (*cls*) and FtsH (*hflB*). Although these truncated ORFs are conserved across various TLPQC-2s, it is unknown whether they are transcribed and expressed or what function they might serve. TLPQC-2 islands were observed on both chromosomal and plasmid locations and were predominantly found in members of the *Enterobacteriaceae*, including *Escherichia*, *Obesumbacterium*, *Klebsiella*, *Cronobacter*, *Salmonella*, and *Yersinia* (Fig. 5) (1–3). Bacterial species that possess TLPQC-2 are of great interest to food industry and human health communities, as many are relevant to food safety such as *Obesumbacterium proteus* as brewery yeast contaminants (34) and *C. sakazakii* as infant formula contaminants (1). Given that a number of TLPQC-2 loci are found on plasmids, the potential exists for transmission and subsequent conferring of heat resistance to foodborne pathogens. In *S*. Senftenberg, the presence of the two TLPQCs on a multireplicon conjugative plasmid highlights the potentially mobile nature of these environmental stress resistance determinants.

The third lineage, TLPQC-3, is the shortest of the three loci (mean length of 6.5 kb), and composed only of the ORFs that encode a heat shock protein, the Clp protease, a truncated version of cardiolipin synthase, the Zn-dependent protease, and the periplasmic serine protease (Fig. 5). Found primarily within *Klebsiella pneumoniae* plasmids, this TLPQC has been associated with the emerging multidrug-resistant *K. pneumoniae* ST16 clone (5, 35). The Clp protease in TLPQC-3 has been characterized and was named ClpK, sharing 98.3% amino acid identity with the pSSE ATCC-43845 ClpG_G1_ (3). The high degree of conservation of the core elements within these islands underscores their apparent importance to host fitness.

### Conclusions.

We describe the presence of two broadly disseminated but uncommon TLPQCs present in the important pathogen *Salmonella enterica*. The discovery of two TLPQCs in *S*. Senftenberg ATCC 43845 underscores its uniqueness and suggests a likely mechanism for the unusually thermotolerant phenotype repeatedly observed for this strain (9–13). As this strain of *S*. Senftenberg has been maintained in collections since at least 1941, *S*. Senftenberg ATCC 43845 is also one of the oldest known isolates to harbor TLPQCs. The presence of TLPQC-1 and TLPQC-2 on a single plasmid raises questions about the selective pressures and genetic events that led to their evolution. As the original source of *S*. Senftenberg ATCC 43845 was powdered eggs, it is tempting to suggest a correlation between the acquisition of the TLPQC loci and exposure to conditions likely encountered in the production of powdered egg. Regardless of how it evolved, the complete genome sequence and methylome data of this unusually thermotolerant strain, as well as the phylogenetic analysis of these broadly distributed islands conferring enhanced mechanisms of protein maintenance, will facilitate future studies on the role of TLPQC loci in extreme heat and desiccation resistance within Gram-negative bacteria important to human health and food safety. In addition, the complete sequence of an IncHI2 R478-like plasmid isolated at the dawn of the era of therapeutic antibiotic use will aid in furthering our understanding of the evolution of resistance plasmids.

## MATERIALS AND METHODS

### DNA isolation and genome sequencing.

S. Senftenberg ATCC 43845 was acquired from ATCC and cultured statically at 37°C in Trypticase soy broth (Becton, Dickinson, Franklin Lakes, NJ) for 18 to 20 h. DNA was purified using Qiagen genomic-tip 100/G columns and the DNeasy blood and tissue DNA isolation kit (Qiagen, Valencia, CA) using the manufacturer’s recommended protocol. Sequencing libraries were prepared following the recommended Pacific Bioscience protocols. Single molecule real-time sequencing (SMRT) was performed using P6/C4 chemistry on a Pacific Bioscience (PacBio) RS II instrument (Pacific Biosciences, Menlo Park CA), resulting in average subreads of >7 kbp and mean coverage of 172.59×. The HGAP3 protocol in smrtanalysis v2.3 was used to assemble sequence reads and polish the contig. To circularize the contigs, a self/self dot plot of the contig sequences was generated in Geneious 9.1.5 (Biomatters Ltd., New Zealand) and used to identify duplicated sequence at the contig ends. The duplicated sequence was trimmed to generate a circularized sequence (36). OriFinder was used to determine the origin of replication and to reset base position 1 of the chromosome. The position of circularization was confirmed by mapping all the reads to the renumbered contig using the resequencing protocol in smrtanalysis v2.3, which also provided a second round of polishing via the included quiver routine. Genome and plasmid sequence data were annotated using the NCBI Prokaryotic Genome Annotation Pipeline and deposited into GenBank (GenBank accession numbers CP016837 and CP016838, respectively).

### Genome analysis.

The sequences of the thermoresistance cluster originally identified in *C. sakazakii* (GenBank accession number FR714908) (1) and the thermotolerance loci in *S*. Senftenberg were used to identify related islands in the GenBank nr database using BLASTn. Genomes with strong similarity to these sequences were identified from 20 genera, and genome sequences were used to extract 91 additional thermotolerance islands by manual inspection to determine the island boundaries (Table 2). Sequence inspection and manipulation were performed in Geneious vR9.1.5. Manual inspection included BLAST comparisons of flanking mobile element genes, which revealed little homology between the different TLPQCs so these flanking sequences were trimmed and discarded to define the boundaries of the TLPQC. The extracted TLPQCs were aligned by MAFFT (37) in Geneious 9.1.5, and the resulting aligned sequences were analyzed by jModelTest 2 to determine the parameters for MrBayes (36, 38, 39). The best model for the full-aligned sequence of the TLPQC was determined to be TRN+I+G (Tamura-Nei model with invariant sites and discrete gamma distribution) using the Akaike information criterion (40). MrBayes 3.2.6, utilized as a Geneious plug-in, was used to calculate a Bayesian inference tree of the various TLPQCs. As the TRN+I+G model is not possible in MrBayes 3.2.6, the GTR+I+G model (generalized time reversible model with invariant sites and discrete gamma distribution) was used, as overparameterizeration gives substantially less bias than underparameterizeration (41), and 3,000,000 iterations were performed with these defined parameters. The first 25% of the iterations were discarded as burn-in, as the Markov chains have not reached stationary and may alter the final result. Visualization of the tracer output and examining the standard deviation of the split frequencies were used to assess the quality of the tree. As the standard deviation of the split frequencies reached only <0.04, the chains have truly not converged but were acceptable, given the extensive computing resources to run the calculations. Additional maximum likelihood trees generated by PhyML (42) gave further support for the Bayesian consensus tree (not shown). Representatives of TLPQCs derived from the Bayesian phylogenetic tree were extracted and visualized in an Easyfig BLASTn comparison (43). CGView Server was used to visualize chromosome and plasmid sequence data with default tBLASTx settings (44).

### Base modification analysis.

Genome analysis of nucleotide base modifications were detected using the RS_Modification Motif_Analysis.1 protocol in smrtanalysis v2.3 (Pacific Biosciences, Menlo Park, CA) with the default threshold quality value (QV) of 30. The resulting motif_summary.csv files were uploaded to the restriction enzyme database (REBASE) and assessed for the presence of novel restriction modification systems (30). Additionally, kinetic Qmod values [defined by the log-transformed *P* value from the *t* test, −10log(*P* value)] with a threshold value of 55 were extracted and processed by BaseModFunctions v2.1.R (45) for visualization in Circos (46). The threshold value of 55 was based on the bimodal distribution of modification values observed in the kinetic detection map. Base modification data have been uploaded to GenBank.

### Accession number(s). 

Accession numbers for the strains used in this study can be found in Table 2.

## References

[1] Gajdosova J, Benedikovicova K, Kamodyova N, Tothova L, Kaclikova E, Stuchlik S, Turna J, Drahovska H 2011 Analysis of the DNA region mediating increased thermotolerance at 58 degrees C in Cronobacter sp. and other enterobacterial strains. Antonie Van Leeuwenhoek 100:279–289. doi:10.1007/s10482-011-9585-y. 21567153

[2] Mercer RG, Zheng J, Garcia-Hernandez R, Ruan L, Gänzle MG, McMullen LM 2015 Genetic determinants of heat resistance in Escherichia coli. Front Microbiol 6:932. doi:10.3389/fmicb.2015.00932. 26441869 PMC4563881

[3] Bojer MS, Struve C, Ingmer H, Hansen DS, Krogfelt KA 2010 Heat resistance mediated by a new plasmid encoded Clp ATPase, ClpK, as a possible novel mechanism for nosocomial persistence of Klebsiella pneumoniae. PLoS One 5:e15467. doi:10.1371/journal.pone.0015467. 21085699 PMC2976762

[4] Williams TL, Monday SR, Edelson-Mammel S, Buchanan R, Musser SM 2005 A top-down proteomics approach for differentiating thermal resistant strains of Enterobacter sakazakii. Proteomics 5:4161–4169. doi:10.1002/pmic.200401263. 16196092

[5] Bojer MS, Hammerum AM, Jørgensen SL, Hansen F, Olsen SS, Krogfelt KA, Struve C 2012 Concurrent emergence of multidrug resistance and heat resistance by CTX-M-15-encoding conjugative plasmids in Klebsiella pneumoniae. APMIS 120:699–705. doi:10.1111/j.1600-0463.2012.02885.x. 22882258

[6] Dlusskaya EA, McMullen LM, Gänzle MG 2011 Characterization of an extremely heat-resistant Escherichia coli obtained from a beef processing facility. J Appl Microbiol 110:840–849. doi:10.1111/j.1365-2672.2011.04943.x. 21219555

[7] Lee C, Wigren E, Lünsdorf H, Römling U 2016 Protein homeostasis—more than resisting a hot bath. Curr Opin Microbiol 30:147–154. doi:10.1016/j.mib.2016.02.006. 26974352

[8] Lee C, Wigren E, Trček J, Peters V, Kim J, Hasni MS, Nimtz M, Lindqvist Y, Park C, Curth U, Lünsdorf H, Römling U 2015 A novel protein quality control mechanism contributes to heat shock resistance of worldwide-distributed Pseudomonas aeruginosa clone C strains. Environ Microbiol 17:4511–4526. doi:10.1111/1462-2920.12915. 26014207

[9] Ng H, Bayne HG, Garibaldi JA 1969 Heat resistance of Salmonella: the uniqueness of Salmonella senftenberg 775W. Appl Microbiol 17:78–82. 5774764 10.1128/am.17.1.78-82.1969 PMC377616

[10] Davidson CM, Boothroyd M, Georgala DL 1966 Thermal resistance of Salmonella senftenberg. Nature 212:1060–1061. 21090498 10.1038/2121060a0

[11] Liu TS, Carlson VL, Snoeyenbos GH 1968 Thermal resistance of smooth and rough derivatives of Salmonella senftenberg 775 W. Poult Sci 47:1155–1162. 5725357 10.3382/ps.0471155

[12] Liu TS, Snoeyenbos GH, Carlson VL 1969 Thermal resistance of Salmonella senftenberg 775W in dry animal feeds. Avian Dis 13:611–631. 5812092

[13] Winter AR, Stewart GF, McFarlane VH, Solowey M 1946 Pasteurization of liquid egg products. III. Destruction of Salmonella in liquid whole egg. Am J Public Health Nations Health 36:451–460. PMC1625795 18016346

[14] Bornstein SS, Saphara I, Strauss L 1941 Frequency of occurrence of Salmonella species. J Infect Dis 69:59–64.

[15] Solowey M, McFarlane VH, Spaulding EH, Chemerda C 1947 Microbiology of spray-dried whole egg; incidence and types of salmonella. Am J Public Health Nations Health 37:971–982. PMC1623882 18016587

[16] McCoy JH 1975 Trends in salmonella food poisoning in England and Wales 1941–72. J Hyg (Lond) 74:271–282. 1054731 10.1017/s0022172400024347 PMC2130381

[17] Timme RE, Pettengill JB, Allard MW, Strain E, Barrangou R, Wehnes C, Van Kessel JS, Karns JS, Musser SM, Brown EW 2013 Phylogenetic diversity of the enteric pathogen Salmonella enterica subsp. enterica inferred from genome-wide reference-free SNP characters. Genome Biol Evol 5:2109–2123. doi:10.1093/gbe/evt159. 24158624 PMC3845640

[18] Arndt D, Grant JR, Marcu A, Sajed T, Pon A, Liang Y, Wishart DS 2016 PHASTER: a better, faster version of the PHAST phage search tool. Nucleic Acids Res 44:W16–W21. doi:10.1093/nar/gkw387. 27141966 PMC4987931

[19] Doublet B, Golding GR, Mulvey MR, Cloeckaert A 2008 Secondary chromosomal attachment site and tandem integration of the mobilizable Salmonella genomic island 1. PLoS One 3:e2060. doi:10.1371/journal.pone.0002060. 18446190 PMC2297512

[20] Staehlin BM, Gibbons JG, Rokas A, O’Halloran TV, Slot JC 2016 Evolution of a heavy metal homeostasis/resistance island reflects increasing copper stress in enterobacteria. Genome Biol Evol 8:811–826. doi:10.1093/gbe/evw031. 26893455 PMC4824010

[21] Carattoli A, Zankari E, García-Fernández A, Voldby Larsen M, Lund O, Villa L, Møller Aarestrup F, Hasman H 2014 In silico detection and typing of plasmids using PlasmidFinder and plasmid multilocus sequence typing. Antimicrob Agents Chemother 58:3895–3903. doi:10.1128/AAC.02412-14. 24777092 PMC4068535

[22] Page DT, Whelan KF, Colleran E 2001 Characterization of two autoreplicative regions of the IncHI2 plasmid R478: RepHI2A and RepHI1A(R478). Microbiology 147:1591–1598. doi:10.1099/00221287-147-6-1591. 11390690

[23] Gilmour MW, Thomson NR, Sanders M, Parkhill J, Taylor DE 2004 The complete nucleotide sequence of the resistance plasmid R478: defining the backbone components of incompatibility group H conjugative plasmids through comparative genomics. Plasmid 52:182–202. doi:10.1016/j.plasmid.2004.06.006. 15518875

[24] Cain AK, Hall RM 2012 Evolution of IncHI2 plasmids via acquisition of transposons carrying antibiotic resistance determinants. J Antimicrob Chemother 67:1121–1127. doi:10.1093/jac/dks004. 22334605

[25] Staehlin BM, Gibbons JG, Rokas A, O’Halloran TV, Slot JC 2016 Evolution of a heavy metal homeostasis/resistance island reflects increasing copper stress in enterobacteria. Genome Biol Evol 8:811–826. doi:10.1093/gbe/evw031. 26893455 PMC4824010

[26] Carattoli A 2009 Resistance plasmid families in Enterobacteriaceae. Antimicrob Agents Chemother 53:2227–2238. doi:10.1128/AAC.01707-08. 19307361 PMC2687249

[27] García-Fernández A, Carattoli A 2010 Plasmid double locus sequence typing for IncHI2 plasmids, a subtyping scheme for the characterization of IncHI2 plasmids carrying extended-spectrum beta-lactamase and quinolone resistance genes. J Antimicrob Chemother 65:1155–1161. doi:10.1093/jac/dkq101. 20356905

[28] Fang L, Li X, Li L, Li S, Liao X, Sun J, Liu Y 2016 Co-spread of metal and antibiotic resistance within ST3-IncHI2 plasmids from E. coli isolates of food-producing animals. Sci Rep 6:25312. doi:10.1038/srep25312. 27143648 PMC4855149

[29] Rhoads A, Au KF 2015 PacBio sequencing and its applications. Genomics Proteomics Bioinformatics 13:278–289. doi:10.1016/j.gpb.2015.08.002. 26542840 PMC4678779

[30] Roberts RJ, Vincze T, Posfai J, Macelis D 2015 REBASE—a database for DNA restriction and modification. Nucleic Acids Res 43:D298–D299. doi:10.1093/nar/gku1046. 25378308 PMC4383893

[31] Pirone-Davies C, Hoffmann M, Roberts RJ, Muruvanda T, Timme RE, Strain E, Luo Y, Payne J, Luong K, Song Y, Tsai YC, Boitano M, Clark TA, Korlach J, Evans PS, Allard MW 2015 Genome-wide methylation patterns in Salmonella enterica subsp. enterica serovars. PLoS One 10:e0123639. doi:10.1371/journal.pone.0123639. 25860355 PMC4393132

[32] Byrne-Bailey KG, Coates JD 2012 Complete genome sequence of the anaerobic perchlorate-reducing bacterium Azospira suillum strain PS. J Bacteriol 194:2767–2768. doi:10.1128/JB.00124-12. 22535943 PMC3347210

[33] Chistoserdova L, Lapidus A, Han C, Goodwin L, Saunders L, Brettin T, Tapia R, Gilna P, Lucas S, Richardson PM, Lidstrom ME 2007 Genome of Methylobacillus flagellatus, molecular basis for obligate methylotrophy, and polyphyletic origin of methylotrophy. J Bacteriol 189:4020–4027. doi:10.1128/JB.00045-07. 17416667 PMC1913398

[34] Koivula TT, Juvonen R, Haikara A, Suihko ML 2006 Characterization of the brewery spoilage bacterium Obesumbacterium proteus by automated ribotyping and development of PCR methods for its biotype 1. J Appl Microbiol 100:398–406. doi:10.1111/j.1365-2672.2005.02794.x. 16430517

[35] Löhr IH, Hülter N, Bernhoff E, Johnsen PJ, Sundsfjord A, Naseer U 2015 Persistence of a pKPN3-like CTX-M-15-encoding IncFIIK plasmid in a Klebsiella pneumonia ST17 host during two years of intestinal colonization. PLoS One 10:e0116516. doi:10.1371/journal.pone.0116516. 25738592 PMC4349654

[36] Kearse M, Moir R, Wilson A, Stones-Havas S, Cheung M, Sturrock S, Buxton S, Cooper A, Markowitz S, Duran C, Thierer T, Ashton B, Meintjes P, Drummond A 2012 Geneious Basic: an integrated and extendable desktop software platform for the organization and analysis of sequence data. Bioinformatics 28:1647–1649. doi:10.1093/bioinformatics/bts199. 22543367 PMC3371832

[37] Katoh K, Standley DM 2014 MAFFT: iterative refinement and additional methods. Methods Mol Biol 1079:131–146. doi:10.1007/978-1-62703-646-7_8. 24170399

[38] Posada D 2009 Selection of models of DNA evolution with jModelTest. Methods Mol Biol 537:93–112. doi:10.1007/978-1-59745-251-9_5. 19378141

[39] Darriba D, Taboada GL, Doallo R, Posada D 2012 jModelTest 2: more models, new heuristics and parallel computing. Nat Methods 9:772. doi:10.1038/nmeth.2109. PMC4594756 22847109

[40] Akaike H 1974 A new look at the statistical model identification. IEEE Trans Autom Contr 19:716–723.

[41] Lemmon AR, Moriarty EC 2004 The importance of proper model assumption in Bayesian phylogenetics. Syst Biol 53:265–277. doi:10.1080/10635150490423520. 15205052

[42] Guindon S, Dufayard JF, Lefort V, Anisimova M, Hordijk W, Gascuel O 2010 New algorithms and methods to estimate maximum-likelihood phylogenies: assessing the performance of PhyML 3.0. Syst Biol 59:307–321. doi:10.1093/sysbio/syq010. 20525638

[43] Sullivan MJ, Petty NK, Beatson SA 2011 Easyfig: a genome comparison visualizer. Bioinformatics 27:1009–1010. doi:10.1093/bioinformatics/btr039. 21278367 PMC3065679

[44] Grant JR, Stothard P 2008 The CGView Server: a comparative genomics tool for circular genomes. Nucleic Acids Res 36:W181–W184. doi:10.1093/nar/gkn179. 18411202 PMC2447734

[45] Ashby M 2014 Bacterial_Basemod_Analysis: Pacific Biosciences. https://github.com/PacificBiosciences/Bioinformatics-Training/tree/master/basemods.

[46] Krzywinski M, Schein J, Birol I, Connors J, Gascoyne R, Horsman D, Jones SJ, Marra MA 2009 Circos: an information aesthetic for comparative genomics. Genome Res 19:1639–1645. doi:10.1101/gr.092759.109. 19541911 PMC2752132

