# Supplementary material for: Genome Sequence of the Thermotolerant Foodborne Pathogen Salmonella enterica Serovar Senftenberg ATCC 43845 and Phylogenetic Analysis of Loci Encoding Increased Protein Quality Control Mechanisms
Source: mSystems. 2017 Feb 28;2(1):e00190-16. doi: 10.1128/mSystems.00190-16 (PMC5347186; doi:10.1128/mSystems.00190-16)
Supplement: FIG S2 [file sys001172090sf2.pdf]

Supplementary Figure 2.

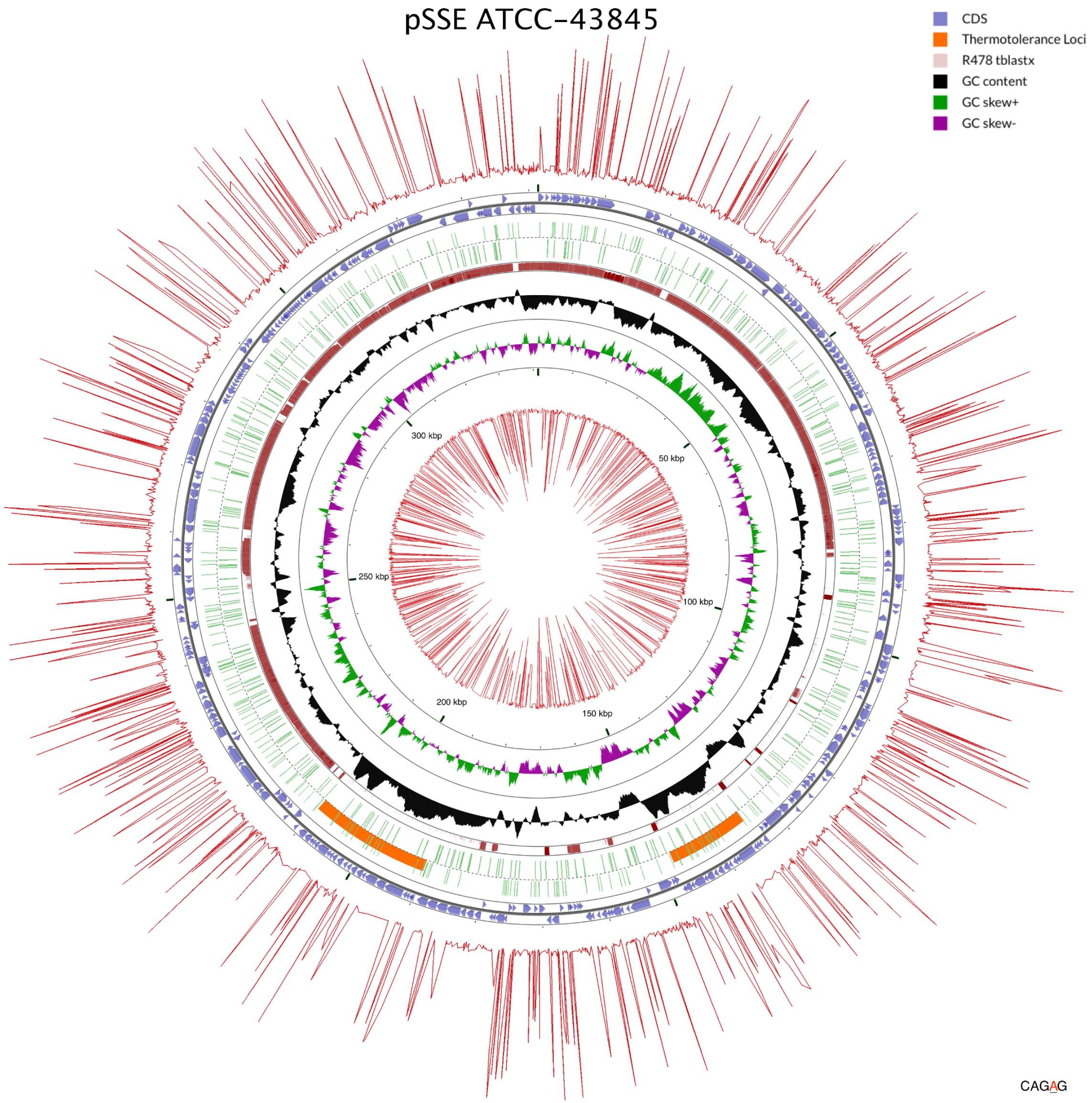

S2. **CAGAG Methylation for pSSE ATCC-43845.** CAGAG methylation motif plotted as previously described in Fig. 2.
